# Deep learning for COVID-19 topic modelling via Twitter: Alpha, Delta and Omicron

**DOI:** 10.1371/journal.pone.0288681

**Published:** 2023-08-01

**Authors:** Janhavi Lande, Arti Pillay, Rohitash Chandra

**Affiliations:** 1 Department of Physics, Indian Institute of Technology Guwahati, Guwahati, Assam, India; 2 School of Sciences, Fiji National University, Suva, Fiji; 3 Transitional Artificial Intelligence Research Group, School of Mathematics and Statistics, UNSW Sydney, Sydney, NSW, Australia; Fiji National University, FIJI

## Abstract

Topic modelling with innovative deep learning methods has gained interest for a wide range of applications that includes COVID-19. It can provide, psychological, social and cultural insights for understanding human behaviour in extreme events such as the COVID-19 pandemic. In this paper, we use prominent deep learning-based language models for COVID-19 topic modelling taking into account data from the emergence (Alpha) to the Omicron variant in India. Our results show that the topics extracted for the subsequent waves had certain overlapping themes such as governance, vaccination, and pandemic management while novel issues aroused in political, social and economic situations during the COVID-19 pandemic. We also find a strong correlation between the major topics with news media prevalent during the respective time period. Hence, our framework has the potential to capture major issues arising during different phases of the COVID-19 pandemic which can be extended to other countries and regions.

## 1 Introduction

The coronavirus pandemic (COVID-19), also known as (SARS-CoV-2) [1–3] gave extensive damage to the world economy, along with widespread infections and about 6.8 million deaths worldwide (as of 18th April 2023). The COVID-19 pandemic had major challenges on mental health and economic consequences during lockdowns [4], due to the closure of businesses and job losses, distribution of rations [5], and administration of vaccines [6]. The *World Health Organisation* (WHO) first reported COVID-19 as a public health crisis in January 2020 and declared it a pandemic in March 2020 [7]. India reported its first case on January 3rd, 2020 with an increase in cases reported from March also declared as India’s first wave [8]. India also reported its first COVID-19 death in March 2020 [9] where a rapid rise in infections led to a nationwide lockdown.

During the first COVID-19 wave in India (March—October 2020), several measures were taken by Indian *Ministry of Health and Family Welfare* and Government of India in collaboration with WHO to prevent and control the spread of the virus within and across states of India [9]. Some measures included personal protection measures, closures of educational institutions, social distancing, closures of historic buildings, transportation measures and local migration [10], sports activities, and biomedical waste disposal [11]. Despite strict implementation, several challenges were faced in controlling the spread of the virus and management of treatment of patients. The challenges included overburdened and understaffed hospitals, lack of medical equipment, and news/information about the pandemic circulated in social media. In the case of India, the return of migrant workers to their respective states also reported an increase in COVID-19 cases [10]. During the first wave, the Government of India announced 20 lakh crore rupees (270 billion USD) package [12] of which 1.7 lakh crore rupees was used for food rations for those most severely affected by the lockdown. In addition, approximately 800 million also benefited from free grain, cash, and cooking gas benefits for three months which addressed the fear of food security in India by WHO [13].

Social media became one of the major platforms to spread information on COVID-19 [14]; with activities such as disseminating information on prevention, control and screening centres. Social media has also been used for reporting death rates, providing advice on lockdowns, and expressing sentiments about COVID-19-related topics [15, 16]. Twitter, a micro-blogging social media platform, was widely used to share and express personal viewpoints and sentiments during the various waves of COVID-19 [15]. Due to publicly available data (tweets) provided by Twitter, it has been used for social media analysis during various events such as elections [17], protests, natural disasters, medical interventions [18], and also the COVID-19 pandemic [19–22].

Topic modelling is a domain in natural language processing (NLP) that is used to detect ‘topics’, analyse them, and classify them according to common patterns and themes emerging across the corpus [23, 24]. It is widely used as a quantitative and qualitative research and analysis tool in the field of bioinformatics [25], management [26], sociology, and media analysis [24]. Other than information retrieval and analysis, topic modelling enables researchers to identify influential individuals and groups on a specific social media platform. It can also be used to detect signs of adverse mental issues such as depression [27, 28]. Topic modelling essentially assists researchers to carry out a smart literature review by categorically compiling literature while avoiding the onerous task of a manual review [29]. The two traditional techniques for topic modelling include Latent Semantic Analysis (LSA) [30] and Latent Dirichlet Allocation (LDA) [31]. LDA assumes that documents are a mixture of topics and each topic is a mixture of words with a certain probability score, whereas LSA uses singular matrix decomposition for reduction of text and extraction of topics [32]. Though these techniques have been widely used over the years for data extraction and analysis, they are not highly suitable for short text documents such as Twitter [33]. Twitter allows users to send and receive 280-character short messages (tweets) making it one of the fastest and most popular data acquisition sources [23].

Researchers have used deep learning models such as *long short-term memory* (LSTM) networks [34] and *bi-directional encoder representative from transformer* (BERT) [35] models that are increasingly becoming popular for language modelling tasks that include sentiment analysis and topic modelling. In a study, Chandra and Krishna [19] implemented LSTM and BERT models for sentiment analysis of Twitter data for the rise of COVID-19 cases in India and reported that the optimistic, annoyed and joking tweets dominated the monthly tweets. Chandra and Ranjan [36] used a BERT-based model with advanced clustering methods for topic modelling by comparing topics of ancient Hindu texts, i.e. the Bhagavad Gita and the Upanishads. In this study, different combinations of BERT-based models with clustering methods were utilised, which provided much better results when compared to LDA. This motivates us to use the same framework to compare topics emerging across the three major peaks for COVID-19 in India, which had a unique set of challenges.

In this paper, we use a deep learning-based language framework for COVID-19 topic modelling taking into account data from COVID-19 emergence, which includes Alpha, Delta and Omicron variants for the three distinct peaks (waves) in India. We use Twitter data from India and also compare it with our earlier works that looked at sentiment analysis of the first wave in India. We note that we refer to the three distant peaks as waves and define their time frame in the methodology section. Our goal is to extract and study the various topics emerging in the three different waves and discuss the relationship to emerging events and issues in the media during the respective time frames. Our goal is to observe how the emerging events affected the topics covered in COVID-19 tweets during the respective time frames. The major contribution of our study is in the application of novel deep learning-based language models for topic modelling during COVID-19 based on Twitter data. We also compare our results with traditional topic models (such as LDA).

The rest of the paper is organised as follows. In Section 2, we provide further details about the methodology which includes data processing and models. In Section 3, we present the results and highlight major observations. Section 4 provides a discussion about the results in relation to emerging events in the respective waves. Section 5 concludes the paper with directions for subsequent research.

## 2 Methodology

### 2.1 COVID-19 waves in India

Although India had a prolonged first wave which lasted almost a year, it recorded lower COVID-19 prevalence in terms of the number of cases and deaths when compared to USA [8]. India had a much lower number of cases and deaths per capita when compared to developed western countries [37]; i.e as of July 12th 2020, India verified 820,916 COVID-19 cases, and 22,123 deaths while the mortality rate of India was 2.69%. In comparison, the USA had 3,097,300 confirmed cases and 132,683 deaths with a mortality rate of 4.28% and the UK had 288,137 cases and 44,650 deaths with a mortality rate of 15.49%. Currently (18th April 2023), India is ranked no. 2 after the USA for the total number of cases and no. 3 after the USA and UK for a total number of deaths. India ranks 145th in 231 countries for per capita deaths in a population of a million (https://www.worldometers.info/coronavirus/). India recorded its second wave with a major and much higher peak of cases that spanned March to July 2021 [8]. In the first wave, the peak was reached with around 97,000 novel daily cases (16th September 2020) [38]. In comparison to the first wave, the second wave had a rapid spread of infections with a peak of more than 400,000 cases reported per day (7th May 2021) from around 9,000 cases (15th February 2021) within three months [38]. The Delta variants, i.e., B.1.617.1 and B.1.617.2 [39] also emerged during this period being one of the major reasons for the rapid increase in cases. This caused serious concerns in the international community and calls for addressing medical support and food insecurity [40]. Afterwards, there was a steady decline in infections mostly attributed to better control and management of the virus and the administration of vaccines [41]. The third wave was recorded in late December 2021 with a major peak of about 306,000 novel daily cases (23rd January 2022), given the Omicron variant [42]. The definition of major variants has been given by the Centre for Disease Control and Prevention (CDC) [43].

### 2.2 Data extraction and processing

We obtained the dataset of tweets originating from India during the COVID-19 first wave from the IEEE Dataport [44] for India. We note that Twitter does not allow the tweets to be shared directly with third parties; hence associated datasets generally feature the tweet identifiers (IDs). We can use tools (known as *hydrator* (https://github.com/DocNow/hydrator) to extract the tweets, which is a time-consuming process due to restrictions given by Twitter to ensure that the data is not misused. We also obtained the dataset of tweet handles (i.e. tweet identifier) for the second and third waves from IEEE Dataport- COV19Tweets [44], which features more than 310 million COVID-19-specific English language tweets. We also published the dataset via Kaggle that we obtained which features major countries and the tweets from emergence to the Omicron variant (till February 2022) [45].

We used the *hydrator* software application to extract the daily tweets worldwide and then separated the India-specific tweets from the global dataset. We have obtained 30,000 tweets per day from India for three selected days a week for the time frame of the second and third wave defined in Table 1. Our data collection methods complied with the *terms and conditions* of Twitter and the IEEE Dataport. We obtained hydrated tweets with the following identifiers with information as given below:

1. *‘coordinates’* represents the geographic location of the tweet as reported by the user or client application.
2. *‘created_at’* gives the UTC date and time that the user account was created on Twitter.
3. *‘hashtags’* represents hashtags that have been parsed out of the tweet.
4. *‘media’* represents a media object representing a single photo, video or animated GIF.
5. *‘urls’* gives web-link provided by the user in association with their profile.
6. *‘favourite_count’* provides the number of Tweets this user has liked in the account’s lifetime. Note that British spelling is used in the field name for historical reasons.
7. *‘id’* provides the integer representation of the unique identifier for a user.
8. *‘in_reply_to_screen_name’* contains the screen name of the original tweet’s author if the tweet is a reply.
9. *‘in_reply_to_status’* contains the integer representation of the original tweet’s ID if the tweet is a reply.
10. *‘lang’*(language) provides language information.
11. *‘country_code’* represents the country.
12. *‘text’* gives name of the hashtag, minus the leading ‘#’ character
13. *‘retweet_count’* provides the number of times the tweet has been retweeted.
14. *‘user_description’* provides the user-defined UTF-8 string describing their account.
15. *‘user_favourite_counts’* provides the number of tweets the user has liked in the account’s lifetime.

**Table 1:** Dataset time-frame defining the three waves with statistical information.

Corpus	Time Frame	# Tweets	# Words	#Cases	#Major Variants	#Mutants
First wave	Mar. 2020—Jan. 2021	159312	2468018	10302012	Alpha	E484Q and E484K
Second Wave	Jan. 2021—Nov. 2021	187531	12274701	24207004	Delta, Delta Plus	K417N
Omicron	Nov. 2021—Feb. 2022	172583	4589488	7652375	Omicron	–

We pre-processed the entire dataset consisting of the three waves, which consists of the following steps.

1. Removal of punctuation and web links (URLs);
2. Removal of mentions in tweets;
3. Conversion of symbols used for emotions (emojis) into text using emoji2text (https://pypi.org/project/emoji2text/);
4. Removal of extra spaces, and lower-casing and removal of stopwords;
5. Lemmatisation process to group similar words using the natural language toolkit (NLTK) library (https://www.nltk.org/).

### 2.3 Models

#### 2.3.1 LDA

LDA is a prominent generative model for discovering the abstract topics that occur in a collection of documents. Hence, LDA has been prominent for topic modelling [46] and has been applied to bioinformatics [25], social media user recommender system [47], and scientific paper recommender system [48]. LDA builds a topic per document model and words per topic model, modelled as *Dirichlet distributions* [31, 49]. LDA allows sets of observations to be explained by unobserved groups; for example, if observations are words collected into documents, it posts that each document is a mixture of a small number of topics. LDA has challenges when it comes to social media such as Twitter due to the short size of the tweets; however, there are some successful applications of canonical LDA [50]. An application used a hierarchical clustering colouring technique based on topics from Sequential LDA for COVID-19 Twitter analysis [51]. Furthermore, the use of informative priors has shown an improvement in the performance of LDA [52].

In LDA, *α* and *η* are proportion parameters and topic parameters, respectively. The topics are given by *β*_1:*K*_, where each *β*_*k*_ is a distribution over the vocabulary. The topic proportion for the *d* the document is *θ*_*d*_, where *θ*_*d*,*k*_ is the topic proportion for topic *k* in document *d*. The topic assignments for the *d* th document are *Z*_*d*_, where *Z*_*d*,*n*_ is the topic assignment for the *n*th word in document *d*. Finally, the observed words for document *d* are *w*_*d*_, where *w*_*d*,*n*_ is the *n*th word in document *d*, which is an element from the fixed vocabulary. The topic assignment *Z*_*d*,*n*_ depends on the per-document topic distribution *θ*_*d*_; and the word *w*_*d*,*n*_ depends on all of the topics *β*_1:*K*_ and the topic assignment *Z*_*d*,*n*_.

#### 2.3.2 GSDMM

The *Gibbs sampling Dirichlet multinomial mixture model* (GSDMM) [53] works well with short text clustering which is a major source of data given social media [54]. Short text clustering is challenging since usually, a single tweet consists of a single topic of unigrams. Mazarura and Waal [55] presented a comparison of the performance of LDA and GSDMM on short texts and reported that the LDA generally outperformed GSDMM on long texts, and on short texts, GSDMM displayed better potential. The model claims to solve the sparsity problem of short text clustering. In LDA, each document is made up of a distribution of topics which has a distribution of words from a document. GSDMM is an extension of LDA which assumes that a document encompasses one topic, which is later updated as more topics are found. This differs from LDA which assumes that a document can have multiple topics in the beginning. Hu et al. [56] showed that GSDMM has better performance than related methods for Web service clustering. The generative process for GSDMM can be expanded for the whole corpus as follows:

1. Randomly choose *T* topic distributions, ${\vec{\beta }}_{t}∼\text{Dirichlet}({\vec{\lambda }}_{\beta })$.
2. Randomly choose a distribution over topics, $\vec{\alpha }∼\text{Dirichlet}({\vec{\lambda }}_{\alpha })$, for the corpus.
3. For each document, $\vec{d}$ where *d* = 1, 2, …, *D*:a) randomly choose a topic $z_{d}∼\text{Multinomial}\left(\vec{\alpha }\right)$; b) randomly choose words $w_{d}∼\text{Multinomial}({\vec{\beta }}_{z_{d}})$.

The collapsed Gibbs sampler was developed based on the following rationale. Whilst conditional distributions can be derived for all the variables since $\vec{z}=\{z_{1},z_{2},\ldots ,z_{T}\}$ is a sufficient statistic for $\vec{\alpha }={\left\{\alpha_{t}\right\}}_{t=1}^{T}$ and $\beta ={\left\{{\vec{\beta }}_{t}\right\}}_{t=1}^{T}$, they can both be calculated from $\vec{z}$. Consequently, if the parameters $\vec{\alpha }$ and *β* are integrated out of the posterior distribution $p(\vec{\alpha },\beta ,\vec{z}|\lambda_{\alpha },\lambda_{\beta })$, we simply need to sample from $\vec{z}$.

#### 2.3.3 BERT for topic modelling via clustering

BERT is a pre-trained language model [35] which is based on Transformers, i.e. encoder-decoder LSTM-based recurrent neural network that features an enhanced memory mechanism known as attention [57]. The encoder generates an encoding that features information about the relevant parts of the inputs, which is passed to the next encoder layer as inputs. The decoder layer does the opposite of the encoder to generate an output sequence, and each encoder and decoder layer makes use of an attention mechanism. A Transformer implements the mechanism of attention by weighting the significance of each part of the input data, which has made them prominent for language modelling tasks [58].

BERT pre-training phase involves semi-supervised learning tasks such as masked language modelling [35, 59, 60] and next sentence prediction [35]. The two BERT variants include *BERT*_*BASE*_ which consists of 12 transformer blocks and a total of 110 million parameters, and *BERT*_*LARGE*_ which consists of 24 transformer blocks with 340 million parameters. Although BERT is pre-trained, it is usually trained further with datasets for specific applications, such as sentiment analysis of COVID-19-related tweets during the rise of novel cases [19], modelling USA 2020 presidential elections [17], and sentiment analysis as a means to compare translations of religious texts [61].

Clustering methods refer to unsupervised machine learning that groups unlabelled data based on a given similarity measure. The goal of clustering algorithms is to assign each data point a label or a cluster identity and several types of clustering algorithms exit with strengths and weaknesses given the type of data [62]. *Hierarchical density-based spatial clustering of application with noise* (HDBSCAN) [63, 64] defines clusters as highly dense regions separated by sparse regions with the goal of finding high probability density regions as clusters. Clustering methods can be used for topic modelling given that a word embedding is obtained from language models. Recently, several topic modelling frameworks used BERT for embedding in combination with clustering methods [65–70]. Furthermore, various BERT-based approaches have been introduced to enhance coherence scores in topic models. BERTopic is a topic modelling technique that uses transformers (BERT-based word embeddings) and class-based TF-IDF to create dense clusters [67]. Top2Vec with a pre-trained BERT model is similar to BerTopic, but it extracts topics based on the cluster’s centroids.

### 2.4 Framework

In topic modelling, a *word* is the basic unit of data which refers to items from the dataset (vocabulary) of size *V* indexed by {1, …, *V*}. A collection of words is known as a *document* which can be denoted as **w** = {*w*_1_, *w*_2_, …, *w*_*N*_} for a sequence of size *N*; where *w*_*i*_ is the *i*^*th*^ word. The collection of *M* documents is known as a corpus denoted by *D* = {**w**_1_, **w**_2_, …, **w**_*M*_} [31].

**Fig 1:**
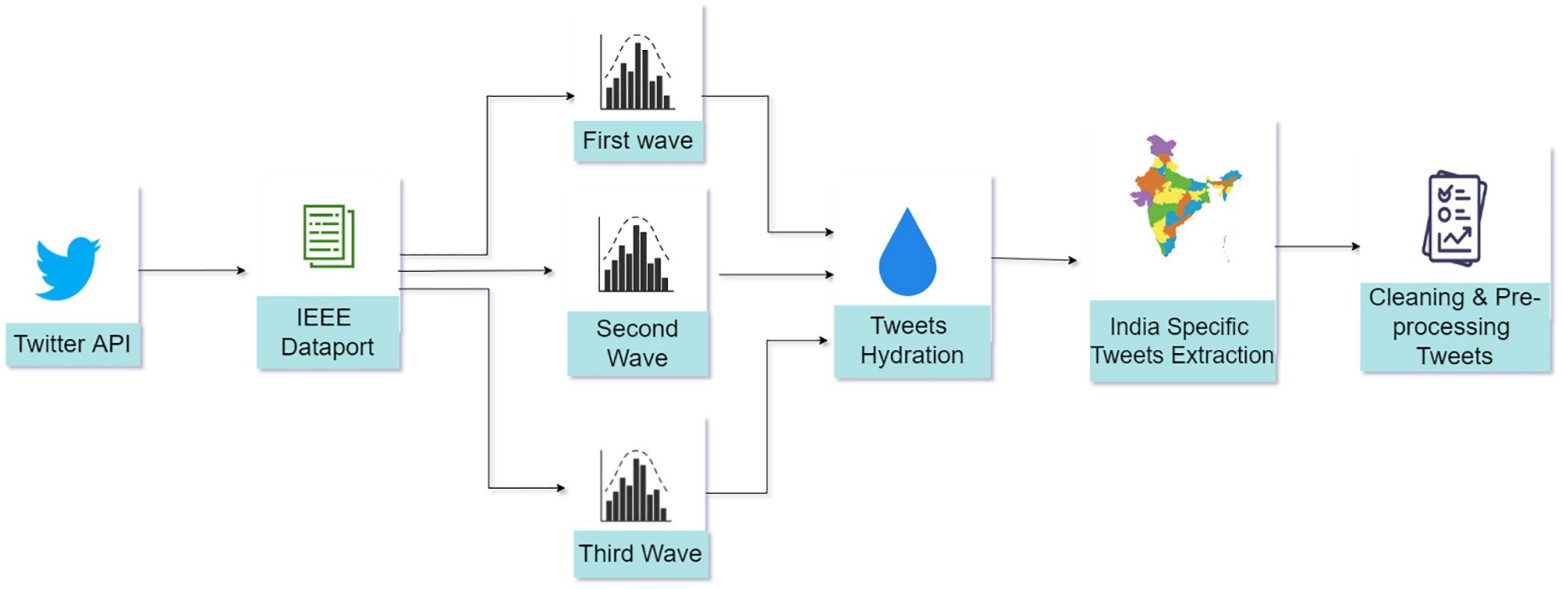
India specific dataset extraction from global COVID-19 tweets dataset.

**Fig 2:**
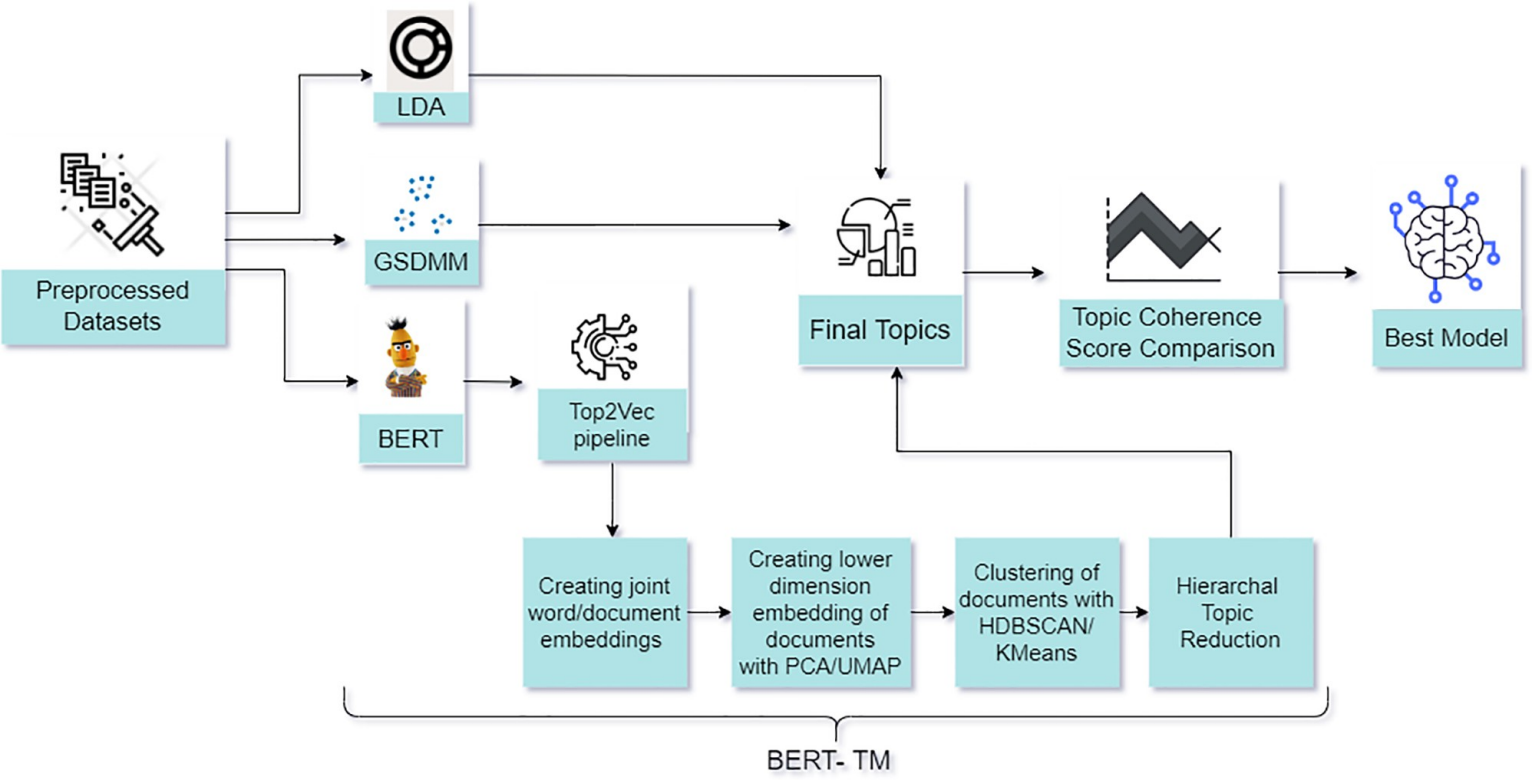
Framework for COVID-19 topic modelling with LDA, GSDMM, BERT-TM using a dataset obtained via Twitter.

We present a framework to employ various machine learning models for topic modeling as shown in Figs 1 and 2. Fig 1 describes the data extraction process of COVID-19 related tweets using a combination of the tweets originating from India during COVID-19 [71] that covers the first wave in India. We obtained the tweets for the second and third waves from our global dataset [46] as described in Section 1.2. As shown in Fig 1, we used location-based extraction to obtain India-specific tweets and pre-processed the tweets of all three waves before modelling.

In Fig 2, our framework begins by training the LDA, GSDMM and BERT-based *Topic2Vec* model. Note that LDA and GSDMM can be directly used for topic modelling and hence the final topics are directly obtained rather than using a word embedding method and clustering algorithm. The original BERT model is computationally intensive for making predictions. Sentence BERT (S-BERT) [72] improved BERT model by reducing computational time to derive semantically meaningful sentence embedding. BERT on its own cannot be used for topic modelling, it only provides a word embedding that would be an input for clustering methods. Hence, our framework employs S-BERT via *BERT*_*base*_ with HDBSCAN which is referred to as the BERT topic model (BERT-TM), hereon. We pass the word embeddings generated by S-BERT to the Top2Vec pipeline where the documents are placed close to other similar documents. Note that S-BERT is embedded within the Top2Vec model in order to obtain word embedding.

Since our S-BERT-based word embedding model has a large vector of features, we need a dimensional reduction method to reduce the features. Hence, we use *uniform manifold approximation and projection (UMAP)* [73] which is a non-linear dimensionality reduction based on Riemannian geometry and algebraic topology. UMAP can be used in a way similar to principal component analysis (PCA) [74] for visualisation and dimensionality reduction of high dimensional data. Chandra et al. [75] evaluated prominent dimensional reduction methods for distinguishing variants of concern based on COVID-19 genome sequences where UMAP was the best method. Although it was a different type of application, motivated by the literature, we use UMAP in our framework for this paper.

In Fig 2, we finally obtain the topic vectors by taking the centroid of document vectors in the original dimension. We then perform the hierarchical topic reduction of the obtained topics in order to assess the similarity between the topics of the three waves. We use *Gensim* [76], an open-source Python library for representing documents as semantic vectors to obtain topic coherence scores. Gensim employs unsupervised learning, which means no target data or human input is necessary, we only need a corpus of plain text documents.

### 2.5 Topic coherence

A topic coherence measure is typically used to evaluate topic models in order to measure their ability to capture topics (i.e. low perplexity) and represent coherent semantic meaning. There are two different strategies for topic coherence measure: 1.) rating, where human evaluators rate the topic quality on a three-point topic quality score, and 2.) intrusion, where each topic is represented by the most frequent words along with an *intruder* which is a word with a very low probability of belonging to the given topic [77]. We use *topic coherence* (TC) [78] as a metric to fine-tune and evaluate different models on a different corpus. The topic coherence metric based on the *normalized pointwise mutual information*(NPMI) correlates really well with the human evaluation and interpretation of the topic coherence [77]. Röder et al. [78] presented a detailed study on the coherence measure and its correlation with the human topic evaluation data.

Note that the NPMI is a step used in topic coherence measure for a pair of words (*w*_*i*_, *w*_*j*_), from the top *N* (set to 50) words of a given topic as given below:

$$\begin{array}{c} \vec{v}\left(W^{\prime }\right)={\left\{\sum_{w_{i}\in W^{\prime }}\text{NPMI}{\left(w_{i},w_{j}\right)}^{\gamma }\right\}}_{j=1,\ldots ,|W|}\\ \text{NPMI}{\left(w_{i},w_{j}\right)}^{\gamma }={\left(\frac{\text{log}\frac{P\left(w_{i},w_{j}\right)+\epsilon }{P\left(w_{i}\right)\cdot P\left(w_{j}\right)}}{-\text{log}\left(P\left(w_{i},w_{j}\right)+\epsilon \right)}\right)}^{\gamma }\\ \phi_{S_{i}}(\vec{u},\vec{w})=\frac{\sum_{i=1}^{\left|W\right|}u_{i}\cdot w_{i}}{\|\vec{u}{\|}_{2}\cdot \|\vec{w}{\|}_{2}} \end{array}$$

where we compute the joint probability of the single word *P*(*w*_*i*_) by the Boolean sliding window approach (window length of *s* set to the default value of 110). We create a virtual document and count the occurrence of the word (*w*_*i*_) and the word pairs (*w*_*i*_, *w*_*j*_), which is then divided by the total number of the virtual documents.

### 2.6 Technical details

In the implementation of our framework, we used a pre-trained S-BERT model, which has been trained on a large corpus of 15 different languages. The model uses *DistilBERT* [79] as the base transformer model, where the output was pooled using an average pooling layer, and a fully connected (dense) layer was used finally to give a 512-dimensional output. We used different combinations of dimensionality reduction techniques and clustering algorithms with pre-trained semantic embeddings to get the final topics for each corpus.

We reduced the embedding dimension to the 5-dimension using UMAP which uses two important parameters, *n_neighbors* and *min_dist* in order to control the local and global structure of the final projection. We used default *min_dist* value of 0.1, *n_neighbors* value of 10 and the *n_components* value of 5. We set the *random-state* to 42 and used *cosine-similarity* as the distance metric. We later used HDBSCAN with parameter *min_samples* = 5, which is used to estimate the probability density of the data points. The *min_cluster_size* defines the smallest grouping size to be considered as a cluster, we set it to 10. Finally, in the remaining two parameters, we used *metric* = *euclidean* and *min*_*samples* = 5.

## 3 Results

### 3.1 Data analysis

**Fig 3:**
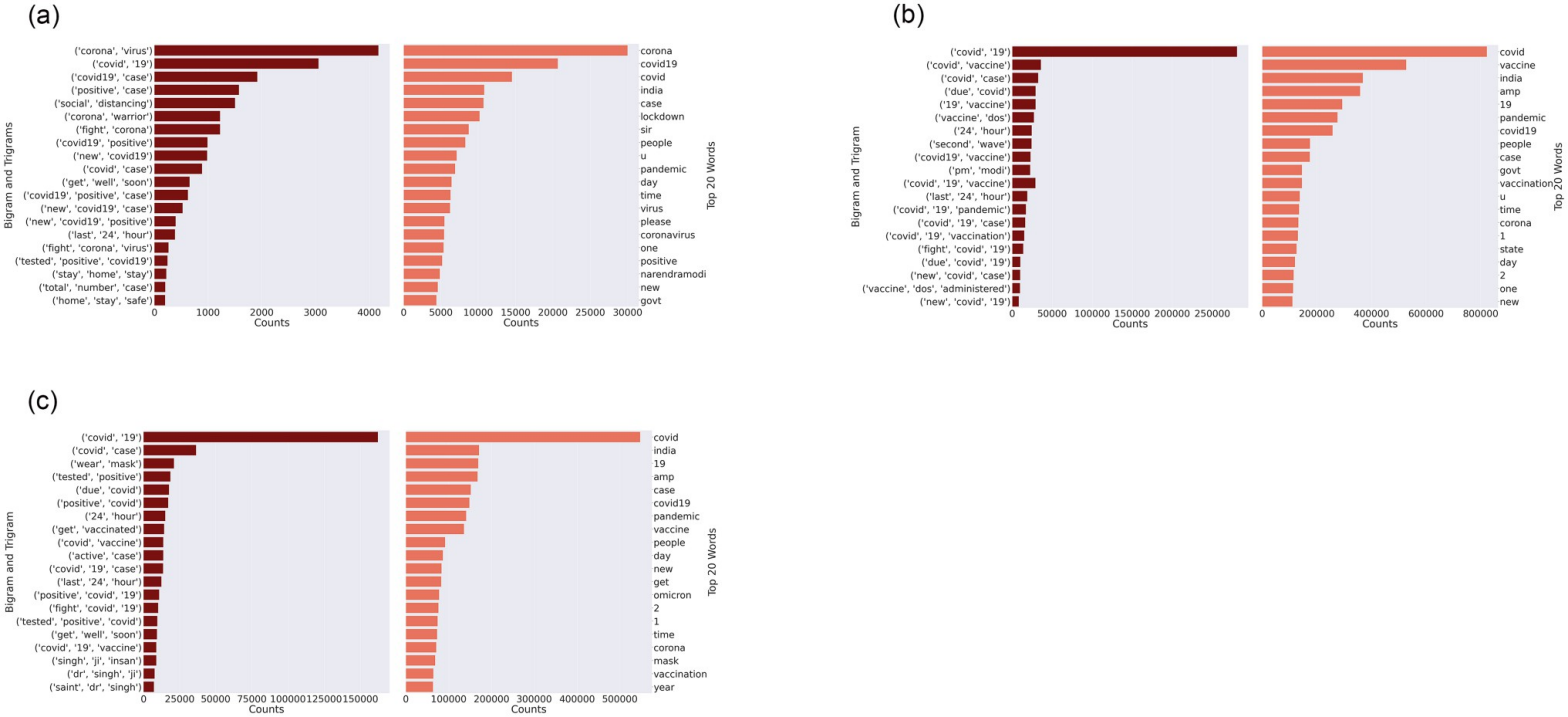
Bigrams and trigrams for the first, second, and third waves of COVID-19 in India.

We first present the bigram and trigram analysis of the three waves of COVID-19 in India. Fig 3—Panel (a) presents the bigrams and trigrams of the first wave. We observe that the bigrams [‘corona’ ‘virus’] and [‘covid’ ‘19’] are more prominent which were used to refer to the pandemic followed by bigrams associated with positive cases and social distancing. Hence, the tweets are centred around understanding the virus and its actual name, the nature of positive cases, and its preventive measures such as social distancing and staying at home. The trigrams further expand the ideas in the bigrams such as [‘covid19’ ‘positive’ ‘case’], with new ones such as [‘get’ ‘well’ ‘soon’] and [‘last’ ‘24’ ‘hour’] which were commonly expressed in media and also been part of official statements of politicians and leaders, not just in India but around the world. Looking at the top 20 words, we find that the word “lockdown” is mentioned slightly fewer times; however, the words mentioned merely refer to the different names of the virus, number of cases, and location (India). This indicates that lockdown as a means to prevent the virus from spreading was highly discussed during the first wave since the lockdowns were harshly implemented then which gained public attention in social media.

Fig 3—Panel (b) shows the bigrams and trigrams for the second wave are mostly similar to the first wave. The bigram [‘second’ wave] and trigram [‘vaccine’ ‘dos’ ‘administration’] point out the major topics that are unique for this dataset. These were discussed in the media during the second wave where the vaccine dosage administration was a major discussion topic. Apart from these, we notice [‘pm’ ‘modi’] which refers to *Prime Minister Narendra Modi* who was active in media briefings with the roll-out of vaccination programme [80]. In terms of the top 20 words, the term ‘vaccine’ is the second most expressed which further shows how important the vaccination process has been during the second wave. In the case of the third wave in Fig 3—Panel (c), the top bigrams and trigrams are similar to the first and the second waves; however, [‘wear’ ‘mask’], [‘get’ ‘vaccinated’], and [‘tested’ ‘positive’ ‘covid’] are some of the key unique ones in the third wave. Apart from these, variations of [‘dr’ ‘singh’ ‘ji’] are also given as trigrams, this could be pointing to Dr Poonam Khetrapal Singh who was at the time the Regional Director of the *World Health Organisation for South-East Asia* [81].

### 3.2 Evaluation of topic modelling methods

We evaluate the respective models by topic coherence of the topics obtained. Table 2 shows the NPMI as a topic-coherence measure for different models (BERT-TM, LDA, and GSDMM) given in the framework shown in Fig 2 for the three different COVID-19 waves in India which were treated as separate datasets (documents). We trained LDA for 200 iterations of default hyper-parameters implemented in the *Gensim* [76] language modelling library. We fine-tuned the number of topic parameters to get the optimal value of NPMI. In Table 2, we observe that in the case of the first wave, BERT-TM gives the highest NPMI score indicating better results. This is followed by GSDMM and LDA, which are closer to each other than BERT-TM. Note that the number of topics extracted by the three models are similar in range (58 and 60).

**Table 2:** NPMI score (higher is better) for the first wave of the COVID-19 pandemic in India.

Model	No. Topics	NPMI
BERT-TM	58	0.69
GSDMM	60	0.39
LDA	58	0.34

Next, we use the best model (BERT-TM) obtained from Table 2 using NPMI and present the topics extracted from the respective documents (waves). Note that the BERT-TM employs dimensional reduction via (UMAP) and clustering via HDBSCAN. In comparison with k-means clustering, the major advantage of HDBSCAN is that it does not require a user-defined value for the number of clusters that corresponds to the number of topics. BERT-TM also uses *hierarchical topic reduction* to reduce the number of topics so that they are more interpretable [68]. Apart from high topic coherence, the other advantage of BERT-TM is that it has major components that can be separately analysed, i.e. we can visualise results from dimensional reduction via UMAP, and also use other methods if needed. Furthermore, we can also visualise results from the clustering component which is implemented by HDBSCAN; hence, better insight into BERT-TM enables it to be an interpretable machine learning model.

### 3.3 Topic modelling: First vs second wave

**Fig 4:**
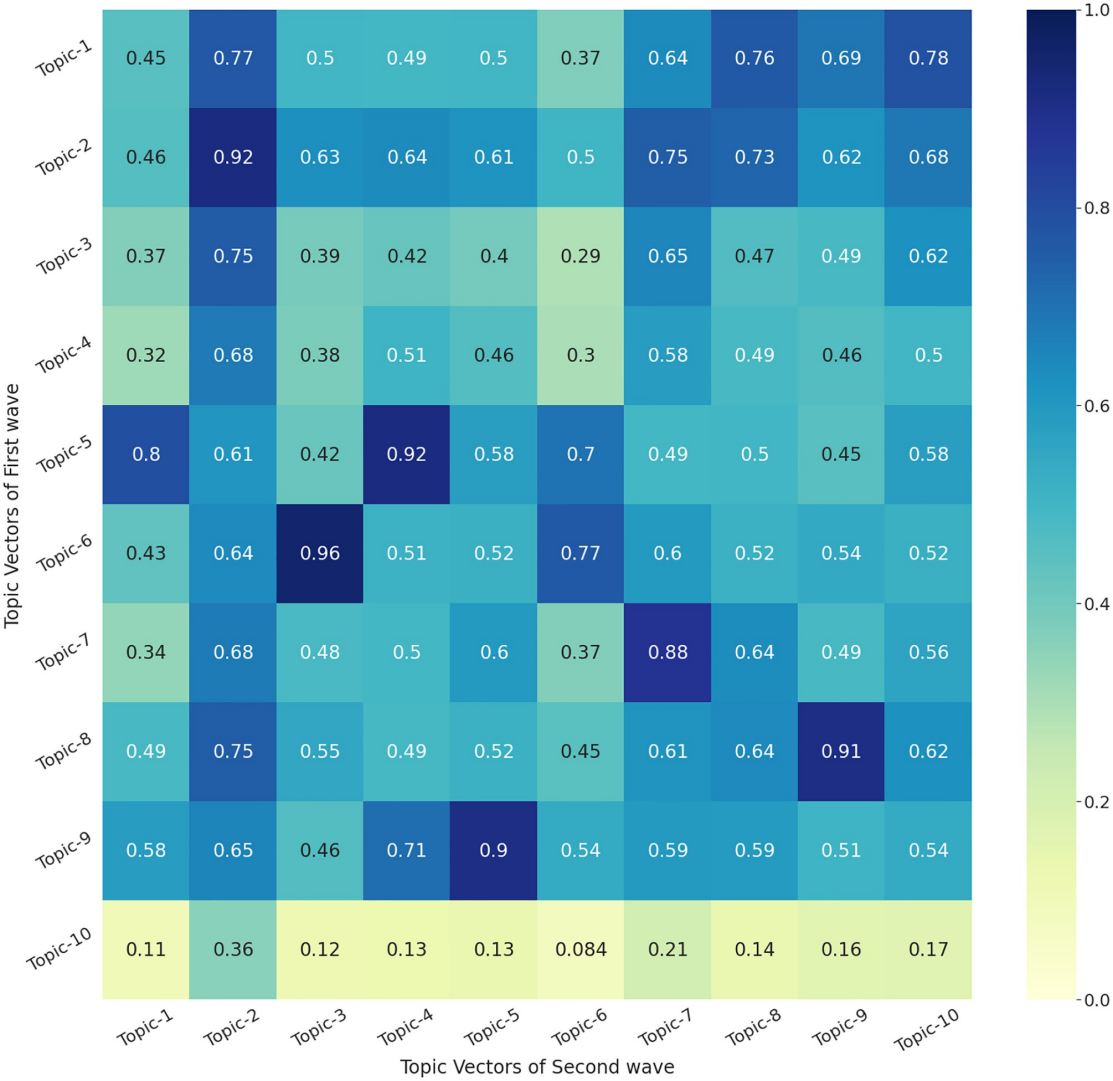
Heatmap showing the similarity score between different topics of First Wave and Second Wave of COVID-19 using BERT-TM.

We carry out further investigations using the BERT-TM which gave the best topic coherence results in the previous section. In order to evaluate the relationship between the first and the second wave of COVID-19, we use the topics obtained to find a similarity matrix and present a heatmap (Fig 4) to establish which topics from the first wave are highly correlated to the topics from the second wave, and vice-versa. The similarity score was computed by cosine similarity. The heatmap represents the cosine similarity of the topic vector obtained by the topic model. Therefore, in each of the topics obtained from BERT-TM, we calculate its similarity with all the topics of the Upanishads and then find the topic with maximum similarity. There are various other measures of similarity score between two vectors; however, the cosine similarity is used widely in the literature [82–84]. One of the major reasons for this is its interpretability. Note that the value of cosine similarity between any two vectors lies between 0 and 1. A value closer to 1 represents perfect similarity and a value closer to 0 represents that they are completely dissimilar. The cosine similarity between any two vectors *U* and *V* is represented by Eq 1. Since the topic vector contains contextual and thematic information about a topic, the similarity score gives us the extent of the closeness of the themes and topics of the COVID-19 waves.

$$\begin{array}{c} \text{Sim(U,}\;\text{V)}=\text{cos}\left(\theta \right)=\frac{U\cdot V}{∥U∥∥V∥} \end{array}$$ (1)

Next, we evaluate and discuss similar relationships both quantitatively using a mathematical formulation and also qualitatively by looking at the topics generated by our models as shown in Table 3, and Fig 4, the vertical axis of the heatmap shows the topics of the first wave while the horizontal axis of the heatmap represents the topics of the second wave. We use the topics obtained to find a similarity matrix in Table 3 where several topics in the first wave are similar to the topics of the second wave with more than 90% similarity.

**Table 3:** Similarity score showing the inter-topic comparison between top 20 keywords of First Wave and Second Wave in India.

Topics of First Wave(top 20 words)	First Wave Topic ID	Most Similar topics in Second Wave	Sec. Wave Topic ID	Score
lockdown,locked,lockdowns,lockdownindia,lockdow,blocked,lock,unlock,lack,prevent,daylockdown,closed,delayed,unemployment,unlocked,badly,over,hence,rather,suffered	topic—1	pandemic,pandemics,catastrophic,crisis,disaster,catastrophe,epidemic,disasters,crises,recession,dengue,worrisome,panic,rising,disastrous,outbreak,collapse,suffered,outbreaks,collapsed	topic -10	0.78
kejriwal,amitabhbachchan,suspected,lakh,tested,suffered,examination,bharat,haryana,hence,today,ahmedabad,jharkhand,amitabh,mukherjee,gandhi,recently,lakhs,chhattisgarh,coronaindia	topic—2	covidisnotover,kejriwal,murshidabad,covidindia,allegedly,breakingnews,ripmilkhasingh,covidsecondwave,covidvaccine,shameonmpgovt,chiyaanvikram,covid,kharcha,kabir,suffered,closely,lakh,seized,whereas,arrestramdev	topic—2	0.91
rather,hence,pathetic,thane,facepalming,toh,amitabhbachchan,worry,than,suspected,which,instead,hdfc,meant,fm,shd,means,suffered,wfh,amitabh	topic—3	covidisnotover,kejriwal,murshidabad,covidindia,allegedly,breakingnews,ripmilkhasingh,covidsecondwave,covidvaccine,shameonmpgovt,chiyaanvikram,covid,kharcha,kabir,suffered,closely,lakh,seized,whereas,arrestramdev	topic—2	0.75
coronaupdate,coronaupdates,corona,coronawarriors,coronaindia,coronalockdown,coronavaccine,coronapandemic,coronil,coron,coronavirus,king,colony,coro,skull,covaxin,covid,chaos,covidiots,covidwarriors	topic—4	covidisnotover,kejriwal,murshidabad,covidindia,allegedly,breakingnews,ripmilkhasingh,covidsecondwave,covidvaccine,shameonmpgovt,chiyaanvikram,covid,kharcha,kabir,suffered,closely,lakh,seized,whereas,arrestramdev	topic—2	0.67
coronavirus,chinesevirus,chinavirus,uhanvirus,vaccine,vaccines,vaccination,virus,viruses,viruse,quarantined,viral,flu,epidemic,viru,coronavaccine,infected,infect,infectious,quarantine	topic—5	vaccineshortage,vaccineforall,vaccinemaitri,vaccinating,vaccinefor,vaccinezehad,vaccinated,vaccineswork,vaccines,vaccination,vaccine,vaccinate,vaccinat,vaccin,vaccinations,vaccinequity,vaccinateindia,vacci,unvaccinated,getvaccinated	topic—1	0.91
indian,india,hindu,indians,hindustan,bharat,kejriwal,hindus,hindi,indi,crore,pakistan,gandhi,bangladesh,lockdownindia,caste,mukherjee,ghaziabad,crores,ahmedabad	topic—6	indiahelp,indian,india,indianarmy,hindu,indians,hindutva,hindustan,bharat,covidindia,kejriwal,vaccinateindia,hindus,hindi,diwali,newindia,healthyindia,nehru,crore,pakistan	topic—3	0.95
appreciate,gratitude,blessed,grateful,appreciated,blessing,thankful,bless,blessings,helping,contribute,contributing,generous,honour,honoured,amitabhbachchan,honourable,thankyou,help,helps	topic—7	shameonmpgovt,ripmilkhasingh,thanking,jharkhand,kejriwal,blessed,kharcha,jammuandkashmir,grateful,diwali,bhadrak,saintramrahimji,radheshyam,appreciate,blessing,murshidabad,ganesh,lakh,gandhi,akshaykumar	topic—7	0.87
governments,government,govt,govts,gov,governance parliament,politicians,authorities,ministers,minister,politician,governor,officials,elected,administration,ruled,corruption,ministry,republic	topic—8	governments,government,govt,govts,gov,governance,corruption,bureaucrats,parliament,politicians,parliamentary,repeal,farmersprotest,corrupt,federalism,politician,thepolitics,governor,administration,democracy	topic—9	0.9
hospitals,hospital,medical,patients,healthcare,clinical,nurse,doctors,nurses,doctorsday,nursing,ambulance,medicine,patient,doctor,clinic,cure,cured,dr,illness	topic—9	hospitals,medical,hospital,hospitalised,hospitalized,healthcare,hospitalization,hospitalisation,ambulance,patients,ambulances,nurse,doctors,medicine,healthyindia,nurses,clinical,medicos,dr,doctor	topic—5	0.9
havoc,sood,coz,hrs,tht,apne,kumar,zany,amit,ble,monday,om,gtu,pic,uttarakhand,jamaat,kerala,kalyan,wuhan,setu	topic—10	covidisnotover,kejriwal,murshidabad,covidindia,allegedly,breakingnews,ripmilkhasingh,covidsecondwave,covidvaccine,shameonmpgovt,chiyaanvikram,covid,kharcha,kabir,suffered,closely,lakh,seized,whereas,arrestramdev	topic—2	0.36

Table 3 presents the top 10 topics with 10 keywords extracted and compares the first wave and second wave in terms of topic similarity score for the COVID-19 pandemic in India. The highest similarity score is between Topic 6 (First Wave) and Topic 2 (Second Wave), followed by Topic 2 (First Wave) and Topic 3 (Second Wave), and Topic 5 (First Wave) and Topic 1 (Second Wave). In comparison to the bigrams and trigrams in Fig 3, we find a level of similarity in the top topics extracted. We notice that the keywords [‘corona virus’], [‘covid’], [‘get well soon’], [‘stay home safe’], [‘narendramodi’], [‘lockdown’] are common with topics extracted from Table 3. The keywords [‘vaccination’], [‘govt’], [‘covid vaccines’], and ‘safe’ are common with the topics extracted from the second wave.

Fig 4 shows that Topic 2 of the Second Wave is highly correlated to several topics of the First Wave (Topic 1, Topic 2, Topic 3 and Topic 8). Furthermore, we find that Topic 3 (Second Wave) is highly correlated to Topic 6 (First Wave), and Topic 4 (Second Wave) is highly correlated to Topic 5 (First Wave). We review these topics with reference to Table 3 and find that the majority of the topics deal with corona, vaccine shortage, government policies, hospitalisation, Prime Minister Narendra Modi, government officials, celebrities, corona updates, etc.

#### 3.3.1 Topics in media

Next, we review the topics with emerging events and reports from the media during the first wave of COVID-19. India observed a nationwide lockdown where the estimated economic cost of the *phase one lockdown* of 21 days (March 25 to April 14, 2020) was estimated to be almost 98 billion dollars [85]. The first wave of lockdown in India was divided into four phases from March until the end of May 2020. In February 2021, India was hit by the largest COVID-19 wave. It was reported in media that people started becoming careless, not wearing masks and not following social distancing, around November to April. This wave caused a rapid surge in cases and deaths and cases began to rise by March 2021, resulting in state-wide lockdowns. In Maharashtra, there were a total of three phases of lockdowns from April to June 2021. Due to large-scale lockdowns, for a period of more than four months, India observed both recession and unemployment (as shown in Table 3, Topic 1).

Indian Yoga Guru, Baba Ramdev made controversial comments about modern medicine and oxygen cylinders (May 2021) [86]. He particularly targeted allopathy medicines which were seen as a competition for Ayurveda medicine promoted by his company that serves as an alternative transitional Indian medicine. There were calls on social media to arrest Baba Ramdev which is evident in Table 3 Second Wave—Topic 2 keyword “arrestramdev”. Indians showed widespread discontent towards government and policies during both the first and second waves, shown by Table 3 First Wave (Topic 8) and Second Wave (Topic 9) with the keywords such as “government”, “corruption” and “bureaucrats”. The patients admitted to the intensive care unit (ICU) during the second wave of the COVID-19 pandemic had significantly higher ICU and hospital mortality [87], whilst both had a high rate of hospitalisation. The keywords ‘coronavirus’, ‘epidemic’, “vaccine” and “vaccineshortage” were widely used throughout the pandemic period to describe COVID-19 as shown in Table 3 (Topics 3, 4,5 from First Wave) and (Topics 2 and 1 from Second Wave). Table 3, Topic 2 keyword (Second Wave) “ripmilkhasingh” refers to the legendary Indian athlete Milkha Singh, also called ‘flying Sikh’ who was a four-time Asian Games gold medallist and 1958 Commonwealth Games champion. He died of COVID-19 in June 2021 and was mourned throughout the country.

India started to experience waves of recession with the coming of the first wave. Over 30% of all industrial goods in India are transported via trains. Therefore, railway freight volumes become an important indicator of economic activity in the country. We note that that many parts of India, including metro cities such as Mumbai and Delhi were under state government-imposed lockdowns. The daily average railway freight volumes in India dropped by 11% in April, according to Indian Railways data [88]. One of the biggest impacts of the lockdowns in 2020 was a sharp rise in unemployment, especially in the unorganised sectors. In April 2020, unemployment in India spiked to 23%. However, as the country reopened, the job market started recovering and by February 2020, the unemployment rate fell to 6.9%. In April 2021, the unemployment rate had gone up to 8.40% [89]. These discussions are evident in Twitter as shown in Table 3, Topic 1 of First Wave and Topic 10 of Second Wave, with keywords about “lockdowns”, “closed”, “delayed”, “collapse”, “outbreaks” and “catastrophe”.

### 3.4 Topic modelling: Second vs Third wave

**Fig 5:**
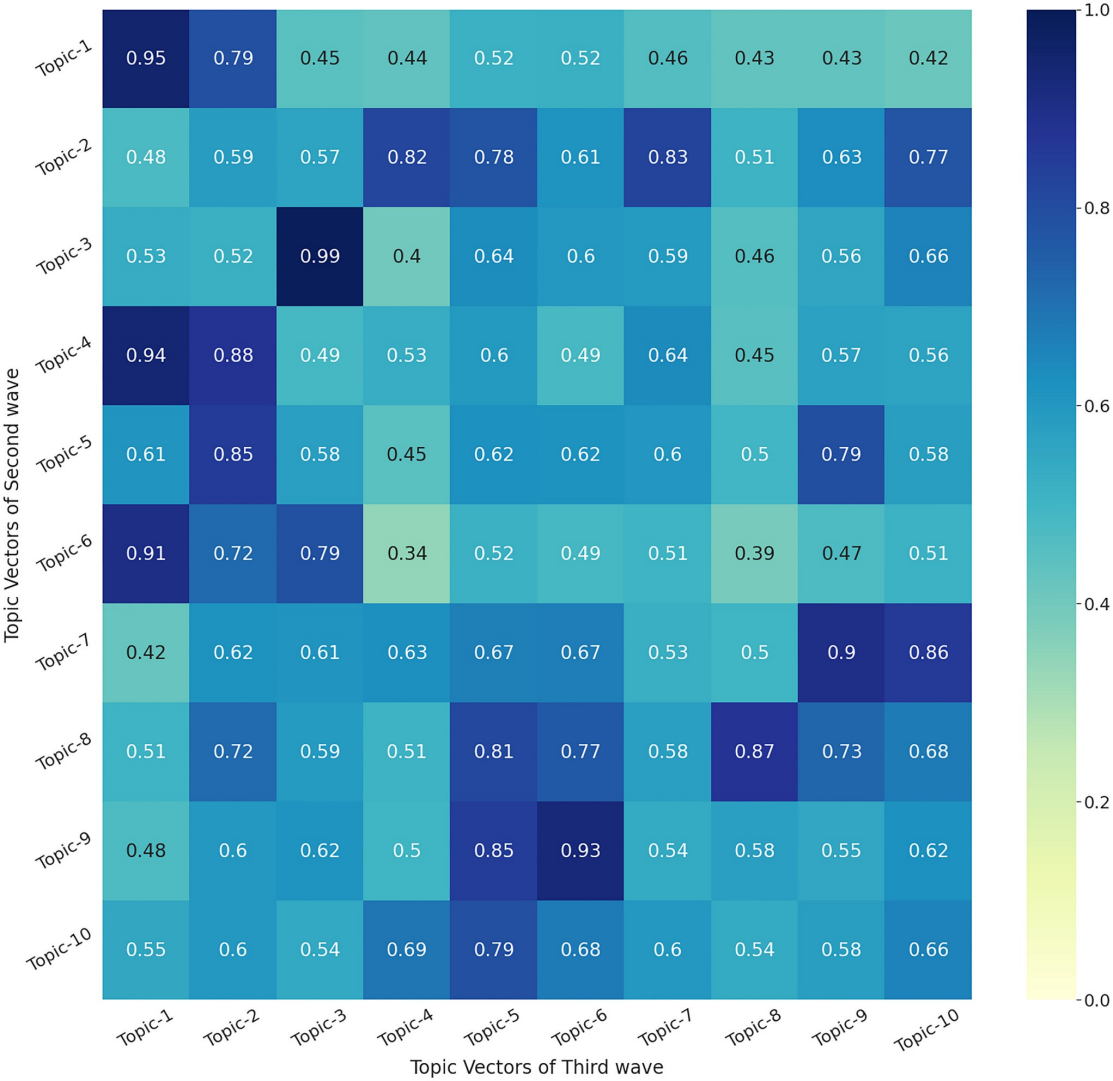
Heatmap showing the similarity score between different topics of Second Wave and Third Wave of COVID-19 using BERT-TM.

We continue with our results from the previous section that compared the first and second waves. We can now compare the second and third waves of COVID-19 in India using the similarity matrix (Fig 5) where the vertical axis shows the topics of the Second Wave while the horizontal axis represents the topics of the Third Wave. The heatmap shows that Topic 1 of the Third Wave is highly correlated with Topics 1, 4 and 6 of the Second Wave. Further, coincidentally Topic 3 of the second wave is highly correlated with Topic 3 of the third wave. The majority of the topics show similarity between vaccine-related issues, viruses, celebrity news, universities, hospitalisation, prayers etc as shown in keywords for respective topics in Table 4.

**Table 4:** Similarity score showing the inter-topic comparison between top 20 keywords of Second Wave and Third Wave in India.

Topics of Second Wave	Topic ID	Most Similar topics in Third Wave	Topic ID	Score
vaccineshortage,vaccineforall,vaccineswork,vaccines vaccinezehad,vaccinemaitri,vaccinefor,vaccinating,vaccinations,vaccine,vaccination,vaccinate vaccinated,vaccinat,vaccin,vaccinequity,vacci,vaccinateindia,unvaccinated,getvaccinated	topic-1	vaccinemandate,vaccines,vaccinating,vaccination,vaccineswork,vaccine,vaccinate,vaccinated,vaccinations,vaccinesuccess,vaccine,vaccinequity,vaccinates,vacci,unvaccinated,getvaccinated,fullyvaccinated,wuhanvirus,ebola,antiviral	topic—1	0.95
covidisnotover,kejriwal,murshidabad,covidindia,allegedly,breakingnews,ripmilkhasingh,covidsecondwave,covidvaccine,shameonmpgovt,chiyaanvikram,covid,kharcha,kabir,suffered,closely,lakh,seized,whereas,arrestramdev	topic-2	newsupdate,latestnews,newstoday,newsupdates,updatenews,middaynews,dailynews,breakingnews,news,indianews,noticias,deaths,cases,newsoftheday,recently,worldcancerday,recent,ommcomnews,fatalities,coronaviruses	topic—7	0.82
indiahelp,indian,india,indianarmy,hindu,indians hindutva,hindustan,bharat,covidindia,kejriwal,vaccinateindia,hindus,hindi,diwali,newindia,healthyindia,nehru,crore,pakistan	topic-3	indianews,indian,indiangovt,india,indianarmy,indias,hindu,indianeconomy,indians,indiamap,indianrailways,indiablooms,hindustani,hindutva,hindustan,bharat,covidindia,bharati,kejriwal,hindi	topic—3	0.99
vaccineshortage,vaccineforall,vaccinemaitri vaccinating,vaccinefor,vaccinezehad,vaccinated,vaccineswork,vaccines,vaccination,vaccine,vaccinate,vaccinat,vaccin,vaccinations,vaccinequity,vaccinateindia,vacci,unvaccinated,getvaccinated	topic-4	vaccinemandate,vaccines,vaccinating,vaccination,vaccineswork,vaccine,vaccinate,vaccinated,vaccinations,vaccinesuccess,vaccin,vaccinequity,vaccinates,vacci,unvaccinated,getvaccinated,fullyvaccinated,wuhanvirus,ebola,antiviral	topic—1	0.94
hospitals,medical,hospital,hospitalised,hospitalized,healthcare,hospitalization,hospitalisation,ambulance,patients,ambulances,nurse,doctors,medicine,healthyindia,nurses,clinical,medicos,dr,doctor	topic-5	vaccinemandate,vaccinated,vaccineswork,vaccines,vaccinating,vaccine,vaccinate,vaccinesuccess,vaccin,vaccination,vaccinations,vaccinequity,getvaccinated,fullyvaccinated,vacci,unvaccinated,vaccinates,ebola,wuhanvirus,immunization	topic—2	0.85
vaccinateindia,vaccineshortage,vaccineforall,vaccines,vaccinezehad,vaccinations,vaccinating,vaccination,vaccinefor,vaccine,vaccineswork,vaccinate,vaccinated,vaccin,vaccinemaitri,vaccinat,vacci,vaccinequity,unvaccinated,getvaccinated	topic-6	vaccinemandate,vaccines,vaccinating,vaccination,vaccineswork,vaccine,vaccinate,vaccinated,vaccinations,vaccinesuccess,vaccin,vaccinequity,vaccinates,vacci,unvaccinated,getvaccinated,fullyvaccinated,wuhanvirus,ebola,antiviral	topic—1	0.9
shameonmpgovt,ripmilkhasingh,thanking,jharkhand,kejriwal,blessed,kharcha,jammuandkashmir,grateful,diwali,bhadrak,saintramrahimji,radheshyam,appreciate,blessing,murshidabad,ganesh,lakh,gandhi,akshaykumar	topic-7	gratitude,blessed,thanking,grateful,blessing,appreciate,goodlucksakhi,bless,thankful,appreciated,wellbeing,condolences,thanked,prayer,fortunately,thankfully,blessings,appreciating,recover,prayed	topic—9	0.9
cancelboardexam,exams,sadly,examdate,exam,failed,unfortunately,failing,caexams,suffered,helpless,volunteers,unemployment,boardexams,didnt,volunteer,unemployed,students,delayed,sorry	topic-8	studentprotest,onlineexams,students,exams,aktuonlineexams,cancelexams,exam,student,colleges,examinations,campus,studying,cancelboardexam,classroom,universities,campuses,graduates,semester,university,classrooms	topic—8	0.87
governments,government,govt,govts,gov,governance,corruption,bureaucrats,parliament,politicians,parliamentary,repeal,farmersprotest,corrupt,federalism,politician,thepolitics,governor,administration,democracy	topic-9	parliament,parliamentary,governmental,governments,government,politicians,corruption,govt,misgovernance,governance,undemocratic,politician,govts,democracy,democratic,democrats,elected,gov,bureaucrats,corrupt	topic—6	0.93
pandemic,pandemics,catastrophic,crisis,disaster,catastrophe,epidemic,disasters,crises,recession,dengue,worrisome,panic,rising,disastrous,outbreak,collapse,suffered,outbreaks,collapsed	topic-10	migrantcrisis,unemployment,overcoming,delayed,disrupted,prevented,crisis,overdue,recession,migrants,boycottmodi,abruptly,slowly,farmersprotest,badly,collapsed,preventing,prevent,overwhelmed,sadly	topic—5	0.79

#### 3.4.1 Topics in media

Next, we discuss how the topics relate to the media coverage of the second wave of COVID-19 in India. The second and third waves in India had major overlapping topics over ‘vaccination’, ‘vaccines’, ‘vaccinework’ as shown in Table 4; Topics 1, 4, and 6 of the Second Wave, and Topics 1 and 2 of the Third Wave. After the second wave, the demand and intake of vaccines started increasing but soon enough due to India’s slow vaccination programme, a shortage was observed. India tried to increase the vaccine doses by banning its export of vaccines for a month [90]. The central government asked the vaccine manufacturers to sell their doses at a lower price and allowed them to sell their vaccines to private healthcare companies at a higher price to compensate. Unfortunately, the private healthcare system didn’t get any incentive for the vaccines from the government. Moreover, the government limited the private hospital’s profit margins. As a result, only large private healthcare companies were interested in the scheme, and the state governments were constantly complaining that they aren’t getting vaccines themselves (as shown in Table 4: keywords in Topic 1 are overlapping both in Second and Third Waves).

Given the possibility of a third wave, in November 2021 the Indian Army increased its medical capacity across the country, while also helping the civilian administration in tackling the coronavirus (as shown in Table 4, Topic 3 of Third Wave). The armed forces registered about 200 COVID-19 cases per day, with the Army alone accounting for about 140 of them. However, most of these cases were mild and haven’t required hospitalisation [91]. The board exams were cancelled [92] as evident with keywords of Topic 8, both in Second and Third Waves of Table 4; “Students should not be forced to appear for exams in such a stressful situation,” said the Indian Prime Minister, adding that all stakeholders need to show sensitivity for students.

The second wave began to witness signs of recession as shown by the keywords, “recession”, “unemployment” and “crisis” (Table 4, Topic 10 in Second Wave and Topic 5 in Third Wave). As per the official data released by the Ministry of Statistics and program implementation, the Indian economy contracted by 7.3% in the April-June quarter of this fiscal year [93]. This was the worst decline ever observed since the ministry had started compiling GDP stats quarterly in 1996. India’s economy shuttered during the lockdown period, except for some essential services and activities. As shops, eateries, factories, and transport services were closed, the lockdown had a devastating impact on slowing down the economy. The informal sectors of the economy have been worst hit by the global pandemic.

### 3.5 Topic modelling: First vs Third wave

**Fig 6:**
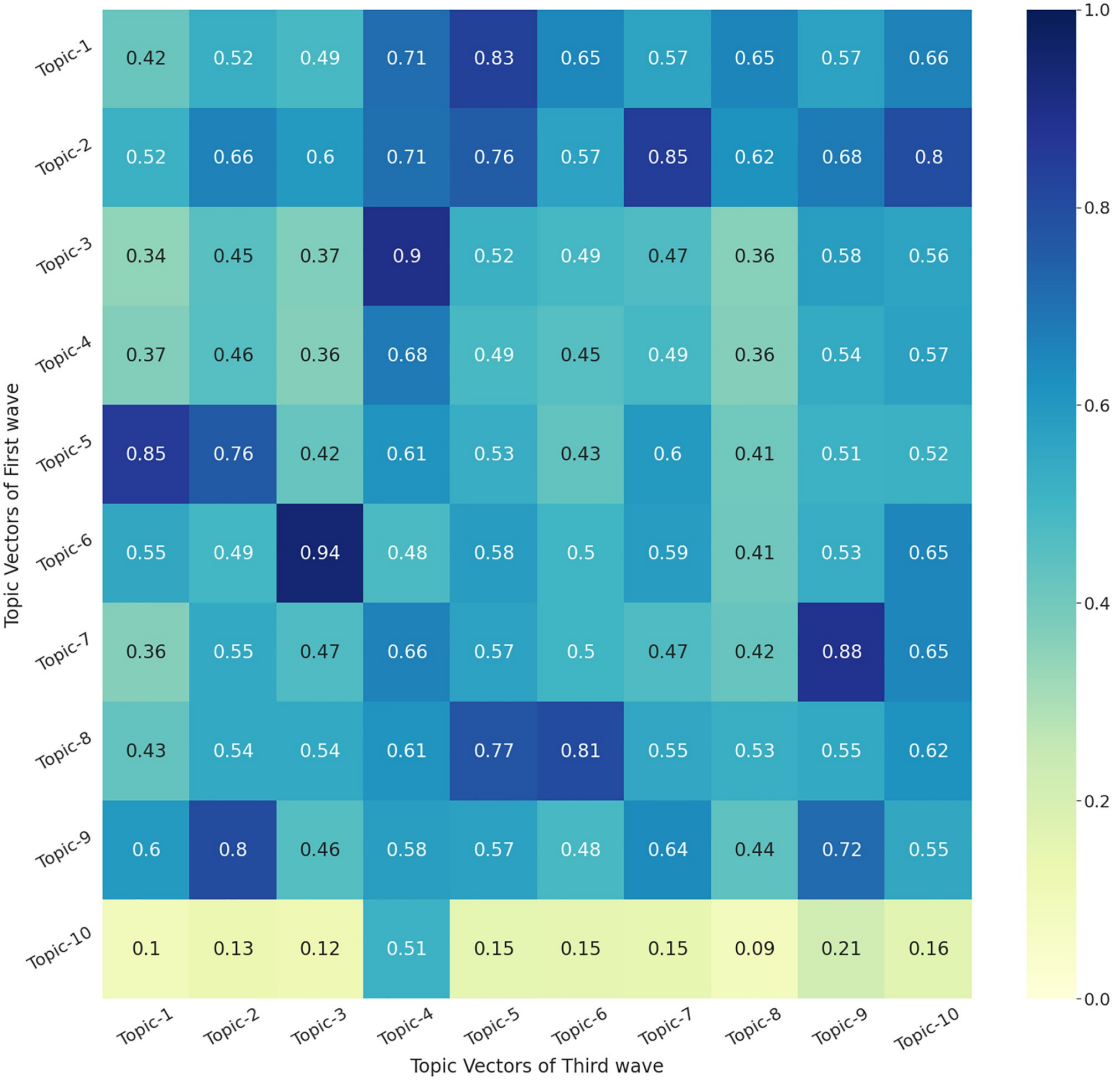
Heatmap showing the similarity score between different topics of First Wave and Third Wave of COVID-19 generated using BERT-TM.

Finally, we compare the first and third waves of COVID-19 in India using the similarity matrix presented (Fig 6). This is to evaluate how the discussion about the COVID-19 pandemic evolved in Twitter and media since the dynamics of the First Wave were very different when compared to the Third Wave.

Fig 6 presents the heatmap that shows that Topic 1 of the Third Wave is highly correlated to Topic 5 of the First Wave. Topic 3 of the Third Wave has the highest correlation with Topic 6 of the First Wave and then there are several other combinations. Table 5 presents topics of the Third Wave vs the First Wave based on the similarity score as shown in the previous section for other wave combinations. We notice that the highest correlated topic between the waves, as pointed out in Fig 6, is given by keywords that have unique terms such as “indian, hindu, kejriwal, pakistan, gandhi, bangladesh, lockdownindia, caste” in First Wave vs “indianarmy, hindu, indianeconomy, indiamap, indianrailways, indiablooms, hindustani, hindutva” (Table 5). This reveals the dynamic nature of COVID-19 and associated topics and the common issues (First and Third Waves) that prevailed in the pandemic such as nationalism, lockdowns, Hinduism, railways, the Indian army and the economy.

**Table 5:** Comparison between top 20 words from the First Wave and Third Wave.

Topics of First Wave	Topic ID	Most Similar topics in Third Wave	Topic ID	Score
lockdown,locked,lockdowns,lockdownindia,lockdow,blocked,lock,unlock,lack,prevent,daylockdown,closed,delayed,unemployment,unlocked,badly,over,hence,rather,suffered	topic -1	migrantcrisis,unemployment,overcoming,delayed,disrupted,prevented,crisis,overdue,recession,migrants,boycottmodi,abruptly,slowly,farmersprotest,badly,collapsed,preventing,prevent,overwhelmed,sadly	topic—5	0.83
kejriwal,amitabhbachchan,suspected,lakh,tested,suffered,examination,bharat,haryana,hence,today,ahmedabad,jharkhand,amitabh,mukherjee,gandhi,recently,lakhs,chhattisgarh,coronaindia	topic -2	newsupdate,latestnews,newstoday,newsupdates,updatenews,middaynews,dailynews,breakingnews,news,indianews,noticias,deaths,cases,newsoftheday,recently,worldcancerday,recent,ommcomnews,fatalities,coronaviruses	topic—7	0.84
rather,hence,pathetic,thane,facepalming,toh,amitabhbachchan,worry,than,suspected,which,instead,hdfc,meant,fm,shd,means,suffered,wfh,amitabh	topic -3	covidisnotover,covidguideline,covidpandemic,covidvaccine,coviddeaths,cooch,covidvaccines,covidguidelines,covid,covidhero,comorbid,covidtest,covovax,covaxin,wbpc,covax,kkundrrasquad,bymygov,sdm,covidpositive	topic—4	0.89
coronaupdate,coronaupdates,corona,coronawarriors,coronaindia,coronalockdown,coronavaccine,coronapandemic,coronil,coron,coronavirus,king,colony,coro,skull,covaxin,covid,chaos,covidiots,covidwarriors	topic -4	covidisnotover,covidguideline,covidpandemic,covidvaccine,coviddeaths,cooch,covidvaccines,covidguidelines,covid,covidhero,comorbid,covidtest,covovax,covaxin,wbpc,covax,kkundrrasquad,bymygov,sdm,covidpositive	topic—4	0.68
coronavirus,chinesevirus,chinavirus,uhanvirus,vaccine,vaccines,vaccination,virus,viruses,viruse,quarantined,viral,flu,epidemic,viru,coronavaccine,infected,infect,infectious,quarantine	topic -5	vaccinemandate,vaccines,vaccinating,vaccination,vaccineswork,vaccine,vaccinate,vaccinated,vaccinations,vaccinesuccess,vaccin,vaccinequity,vaccinates,vacci,unvaccinated,getvaccinated,fullyvaccinated,wuhanvirus,ebola,antiviral	topic—1	0.85
indian,india,hindu,indians,hindustan,bharat,kejriwal,hindus,hindi,indi,crore,pakistan,gandhi,bangladesh,lockdownindia,caste,mukherjee,ghaziabad,crores,ahmedabad	topic -6	indianews,indian,indiangovt,india,indianarmy,indias,hindu,indianeconomy,indians,indiamap,indianrailways,indiablooms,hindustani,hindutva,hindustan,bharat,covidindia,bharati,kejriwal,hindi	topic—3	0.94
appreciate,gratitude,blessed,grateful,appreciated,blessing,thankful,bless,blessings,helping,contribute,contributing,generous,honour,honoured,amitabhbachchan,honourable,thankyou,help,helps	topic -7	gratitude,blessed,thanking,grateful,blessing,appreciate,goodlucksakhi,bless,thankful,appreciated,wellbeing,condolences,thanked,prayer,fortunately,thankfully,blessings,appreciating,recover,prayed	topic—9	0.88
governments,government,govt,govts,gov,governance,parliament,politicians,authorities,ministers,minister,politician,governor,officials,elected,administration,ruled,corruption,ministry,republic	topic -8	parliament,parliamentary,governmental,governments,government,politicians,corruption,govt,misgovernance,governance,undemocratic,politician,govts,democracy,democratic,democrats,elected,gov,bureaucrats,corrupt	topic—6	0.8
hospitals,hospital,medical,patients,healthcare,clinical,nurse,doctors,nurses,doctorsday,nursing,ambulance,medicine,patient,doctor,clinic,cure,cured,dr,illness	topic -9	vaccinemandate,vaccinated,vaccineswork,vaccines,vaccinating,vaccine,vaccinate,vaccinesuccess,vaccin,vaccination,vaccinations,vaccinequity,getvaccinated,fullyvaccinated,vacci,unvaccinated,vaccinates,ebola,wuhanvirus,immunization	topic—2	0.8
havoc,sood,coz,hrs,tht,apne,kumar,zany,amit,ble,monday,om,gtu,pic,uttarakhand,jamaat,kerala,kalyan,wuhan,setu	topic -10	covidisnotover,covidguideline,covidpandemic,covidvaccine,coviddeaths,cooch,covidvaccines,covidguidelines,covid,covidhero,comorbid,covidtest,covovax,covaxin,wbpc,covax,kkundrrasquad,bymygov,sdm,covidpositive	topic—4	0.51

#### 3.5.1 Topics in media

It was reported by the government that over 10 million (one crore) [94] inter-state migrant workers returned home on foot from March to June 2020 during the first wave which had lockdowns which activists called an “under-representation of the scale of the crisis”. The Indian scientists and research institutions were praised with two vaccines indigenously developed and manufactured and had been approved for emergency use in India with a competitive level of efficacy when compared to western counterparts [41, 95]. The world’s largest vaccine drive was underway in the country and was moving forward at a rapid pace with more than 10 million doses administered already by the end of the second wave [96], although there were major challenges in terms of vaccination of rural areas and vaccine hesitancy [97].

### 3.6 Further visualisation

**Fig 7:**
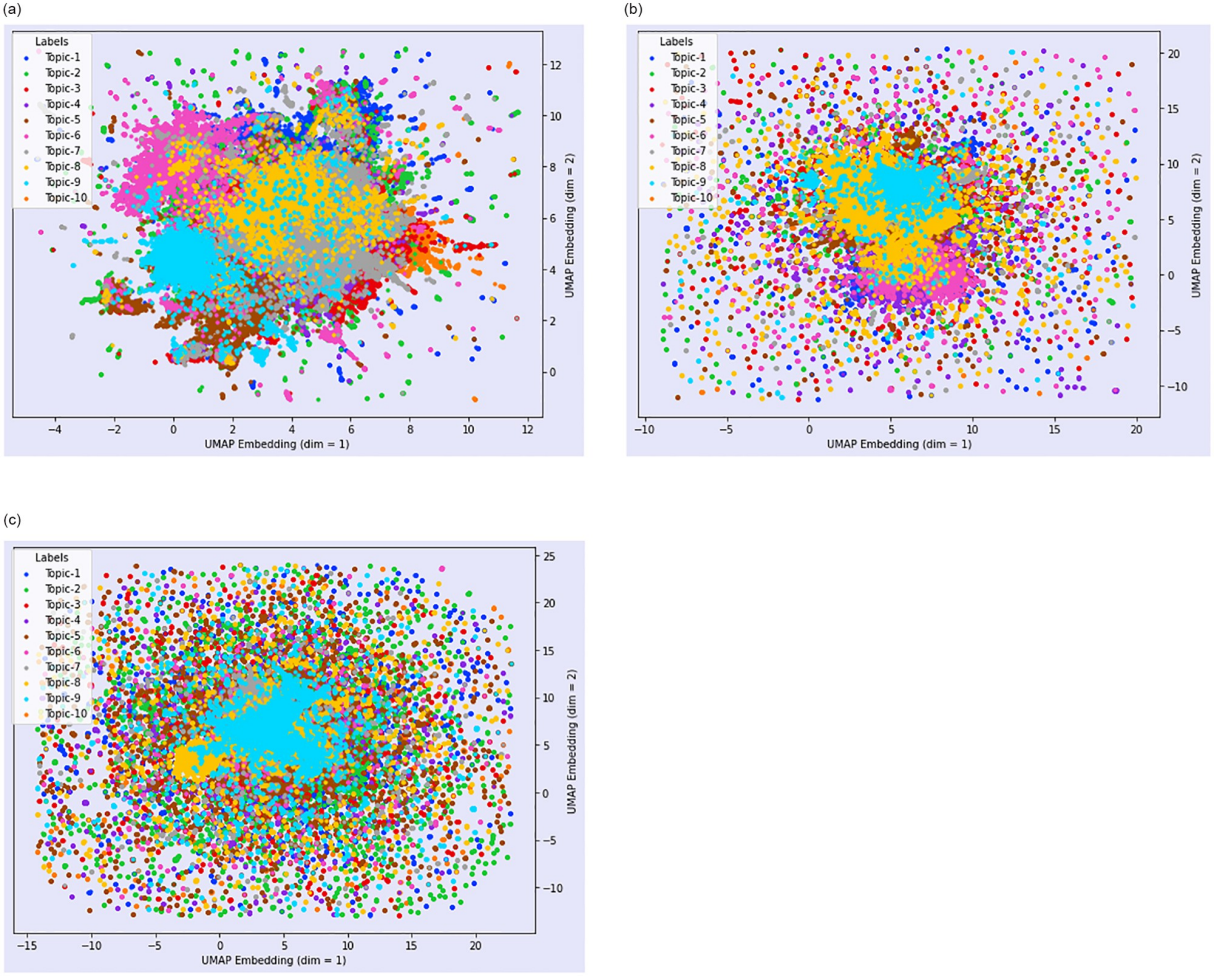
Scatter plot of UMAP embedding of the first, second and third waves after hierarchical topic reduction. (a) First wave (b) Second wave (c) Third wave.

Fig 7 presents a scatter plot of the first two UMAP embedding of the first, second and third COVID-19 waves after implementing hierarchical topic reduction. We reduced the number of topics using hierarchical topic reduction [68]. Since the number of documents and words are different for the different corpus as seen, the number of topics obtained are different for different corpus. We reduced the number of topics to 10 in order to visualize the topic’s semantic space clearly while plotting the semantic space for the different topics obtained by our framework.

In Fig 7 Panel (a), we notice that Topic 9 dominates along with Topic 3 for the first wave. We note that this is for visualisation purposes only and it is not possible to know what exact topic is displayed since we are displaying only the UMAP embeddings. Tables 3 and 4 present the major topics that are linked to Fig 7. In the case of the second wave, Fig 7 Panel (b) shows that Topic 9 largely dominates and finally, for the third wave, we find that Topic 9 dominates. It is unclear what exactly these topics are but if we look at the similarity score from Tables 3 and 4, we can infer that the topics that have large clusters may relate to the topics that have higher similar scores across the waves.

## 4 Discussion

India witnessed a number of major events during the three waves, which included several regional elections spanning 2020—2022 [98], farmers protest [99], and roll-out of vaccines [100]. Our results show that the first, second and third waves observed a variety of overlapping, as well as, distinct topics. India suffered from forced lockdowns and the closure of borders, and its diplomatic relations with other countries also suffered. Although social media played a vital role in the pandemic, there were often alterations in the dissemination of reports from the authorities, resulting in misinformation in social media [101] which had positive and negative impacts [16], which was not constrained to India.

Our results indicate that the major topics during the first wave feature lockdowns, economic crises, school closures, vaccines, government policies, the reaction of the people, death polls, donations, celebrities in India, doctors and hospitals, and religion. The second wave saw a rise in the number of topics related to vaccines such as *Covaxin* with the age group-wise vaccine drive in India [102]. We found that vaccination, hospitalisation and governance were central to the topics from the discussions [103]. We note that the second wave was the most severe in India due to the Delta variant [104] with a higher rate of infections and death rate [8]. In the third wave, with the Omicron variant [42], the topics ranged from vaccination, and governance, to economic recovery which marked the end of the pandemic as restrictions were eased and travel became normal with fewer restrictions. This was mainly because India had a high rate of vaccination by the third wave, which was less severe than the first and second waves as the country was well prepared in medical supplies and management of hospitals [105].

We note that a large effort was made in hydration of the dataset using the Twitter tool known as *hydrator*. Our team manually downloaded and checked the process at regular intervalsalong with segregation of monthly data as well as country-wise, this took more than six months of our time. However, we managed to publish the dataset (password protected to suit Twitter policy) in *Kaggle* [45]. Our data covers the COVID-19 pandemic from March 2020 i.e. from the emergence of COVID-19 capturing major Twitter active countries such as the USA, UK, Brazil, India, Japan, Indonesia, Australia, and Indonesia. In this paper, we restricted the study to India; however, in future works, the study can extend to other countries. It would be interesting to compare the topics emerging from different countries at the different phases of the pandemic.

In terms of limitations, we note that the data source considered was not taken daily, but taken on three selected days of the week. Moreover, it is difficult to apply topic modelling methods to tweets since they are restricted by size and also include everyday language expressions that rely on local regions and are also influenced by regional languages in India. Although the data is sourced in English, we note that most of India has English as a second language and there are a number of regional languages in India. The 2011 Indian census recorded 31 regional Indian languages (such as Hindi, Bengali, Tamil, and Punjabi) had at least one million speakers each [106]; this gives a better picture of the language diversity in India which is a major challenge when it comes to language translation systems [107]. Hence, there would be a bias in expression, with terms that are associated with regional languages according to the Tweet user background. Language translation for Indian languages has been of interest [108] along with speech recognition for Indian languages [109]. Furthermore, the topic coherence score (NPMI) is an approximate measure that can change for different types of documents and we need qualitative studies to further validate the topics. In our study, we validated selected topics extracted using media sources during the different waves of the pandemic. However, this is not a systematic approach and a major challenge of the topic modelling method is the validation of results.

## 5 Conclusions

In this paper, we presented a topic modelling framework for COVID-19 topic modelling in India via Twitter. We first compared BERT-based topic modelling with conventional approaches and found that BERT-based topic modelling performs better in terms of topic coherence. Hence, we used it further to extract topics from the three major waves in India and reviewed the correlation of major topics between the different waves. We reported topics that were distinct for particular waves and also prominent throughout the pandemic. We found a strong correlation between some of the topics qualitatively to news media prevalent at the respective time-frames (waves). Our topic modelling framework provides a systematic methodology for understanding the major topics during COVID-19 in social media that cover governance, vaccination, management, and challenges that included lockdowns and the economy. The framework can be extended to other countries and events to study topics emerging in social media.
